# Haemosporidian Parasites of White-Breasted Waterhens (*Amaurornis phoenicurus*), with a Report and Molecular Characterization of *Haemoproteus gallinulae* in Thailand

**DOI:** 10.3390/ani13122006

**Published:** 2023-06-16

**Authors:** Phirom Prompiram, Kanaporn Poltep, Nattarun Chaisilp, Warunya Chakritbudsabong, Supakit Buamas, Sasitorn Rungarunlert

**Affiliations:** 1 The Monitoring and Surveillance Center for Zoonotic Diseases in Wildlife and Exotic Animals, Faculty of Veterinary Science, Mahidol University, Nakhon Pathom 73170, Thailand; phirom.prm@mahidol.edu (P.P.); kanaporn.pol@mahidol.edu (K.P.); natnapat.cha@mahidol.edu (N.C.); 2 Department of Preclinic and Applied Animal Science, Faculty of Veterinary Science, Mahidol University, Nakhon Pathom 73170, Thailand; warunya.cha@mahidol.edu; 3 Laboratory of Cellular Biomedicine and Veterinary Medicine, Faculty of Veterinary Science, Mahidol University, Nakhon Pathom 73170, Thailand; 4 Clinical Laboratory of Prasu-Arthorn Animal Hospital, Faculty of Veterinary Science, Mahidol University, Nakhon Pathom 73170, Thailand; supakit.bua@mahidol.edu

**Keywords:** AMPHO01 lineage, Gruiformes, *Haemoproteus gallinulae*, lowland, *Plasmodium collidatum*, *Plasmodium elongatum*, Rallidae

## Abstract

**Simple Summary:**

Haemosporidian parasites infect terrestrial vertebrates as well as avian species worldwide. However, for resident birds of the Rallidae family, such as White-breasted Waterhens, few data are available regarding haemosporidian infection. Therefore, this study aimed to detect haemosporidian infection in White-breasted Waterhens, microscopic and genetic examinations of blood samples were performed. Four species of haemosporidians were identified; however, the morphological features of only *Haemoproteus gallinulae* are presented. Phylogenetic analysis of standard DNA sequences revealed a close relationship between *Haemoproteus* species and their classification into the subgenus *Parahaemoproteus*. However, three *Plasmodium* species were found to have a wide range of hosts. The phylogenetic tree of this parasite clearly showed individual clusters. For one of them, the species was not identified; however, it matched a previously reported species that was isolated from mosquitoes. Two of the *Plasmodium* species were identified as well-known, generalist parasites that cause severe diseases in hosts: *P. collidatum* and *P. elongatum*. This study revealed the role of White-breasted Waterhens as carriers of haemosporidian parasites. Our findings provide potential information for further research.

**Abstract:**

Haemosporidian parasites are vector-borne parasites infecting terrestrial vertebrates as well as avian species, such as the White-breasted Waterhen, a Gruiformes waterbird found in lowlands near wetlands and distributed throughout Thailand. However, information regarding haemosporidia infection in this species is lacking. To establish regional information, 17 blood samples were collected from White-breasted Waterhens. Four haemoparasite lineages were identified in six blood samples: *Haemoproteus gallinulae*, *Plasmodium collidatum*, *Plasmodium elongatum*, and an unidentified *Plasmodium* species. *H. gallinulae* was characterized with morphological features in White-breasted Waterhens for the first time; the morphological characteristics were consistent with previous descriptions. *H. gallinulae* was more closely related to *Haemoproteus* species of Passeriformes birds than to those of Gruiformes birds. The *Plasmodium* parasites infecting these White-breasted Waterhens previously caused severe avian malaria in other host species. The unidentified *Plasmodium* species had rarely been documented, although it was reported in the *Culex* vector and was possibly associated with specialist parasites either as host or habitat. Our findings reveal multiple haemosporidian species reflecting the role of this avian host as a carrier of haemosporidians. This study offers species records and molecular materials that may provide critical information for further targeted research into these haemosporidia.

## 1. Introduction

Haemosporidian parasites in all terrestrial vertebrate, likewise in avian species, are vector-borne blood parasites classified into four genera: *Haemoproteus*, *Plasmodium*, *Leucocytozoon*, and *Fallisia* [1]. These parasites are not a frequent cause of death, although the resulting infection can be mild to severe, dependent on the degree of immunity to the parasitic infection [2] and of the infected host species (tolerant or resistant) [3]. Moreover, avian blood parasite infections affect body conditions and growth rate of tail feather, thereby possibly reducing reproductive success in migratory birds and wild passerines [4,5]. However, haemosporidian infection was found to be occasionally highly virulent, such as infections caused by *Plasmodium homocircumflexum*, which further caused anemia and cerebral paralysis and led to the death of experimentally infected canaries [6]. Highly pathogenic infections caused by *P. elongatum* (GRW6) and *P. matutinum* (LINN1) in New Zealand Kiwi (*Apteryx* spp.) have also been reported [7]. Furthermore, infection with haemosporidian parasites contributed to population decline in avian species such as European Turtle Dove (*Streptopelia turtur*) [8] and Skylark (*Alauda arvensis*) [9]. Therefore, a survey of pathogens—expanding on the knowledge of causes of morbidity and mortality—can be implemented to manage and prevent haemosporidian infections.

The White-breasted Waterhen (*Amaurornis phoenicurus*) is a waterbird of the family Rallidae in the order Gruiformes that is resident in south and southeast Asia. In Thailand, these birds are generally found near freshwater in agriculture fields [10] and are considerable an indicator of the successful restoration of wetland biodiversity [11]. In residential regions, a survey of parasites in White-breasted Waterhens previously revealed helminth parasites in Japan and Thailand [12,13] and an unidentified *Plasmodium* parasite in Malaysia [14]. However, the White-breasted Waterhen was found to be an additional host of *Haemoproteus gallinule*, similar to other Rallidae such as Red-legged Crake (*Rallina fasciata*) and Ruddy-breasted Crake (*Porzana fusca*) [15]. *Haemoproteus* parasites have rarely been reported in wild birds of Thailand. Only eight morphospecies have been solely isolated from passerines [16]. Furthermore, in a previous study, *Haemoproteus* parasites of Gruiformes were found to have only four morphospecies, and singular *H. antigonis* was available for molecular detection and identification (barcoding) [17]. A survey of haemosporidian parasites in White-breasted Waterhens can provide potential information on haemosporidia infections in Rallidae; furthermore, it can provide insights into the regional distribution of wildlife parasites in White-breasted Waterhens. Therefore, this survey aimed to detect and describe the haemosporidian parasites found in White-breasted Waterhens based on morphological and molecular examinations.

## 2. Materials and Methods

### 2.1. Study Sites and Sample Collection

White-breasted Waterhens (*Amaurornis phoenicurus*) are a resident bird that frequently forages around lowland paddy fields where abundant with insects, small fish, aquatic invertebrates, and grains. This bird nestled in dry locations and colonized nearly freshwater. During the 2021 dry season, birds were decoy-trapped individually using bird sounds and mist nets in the Salaya suburb, Phutthamonthon district, Thailand (13°48′7″ N, 100°19′18″ E). This location is represented and relates to the White-breasted Waterhen habitat. Seventeen birds were trapped and transported to The Monitoring and Surveillance Center for Zoonotic Diseases in Wildlife and Exotic Animals, Faculty of Veterinary Science, Mahidol University. Approximately 0.5 mL of blood was collected from the brachial vein and a thin blood smear was immediately prepared, allowed to air-dry, and fixed with absolute methanol. Duplicate or triplicate smears were prepared for each bird. The remainder of the blood sample was transferred into a 1.5-mL EDTA tube and stored at −20 °C until DNA extraction was performed. Then, all birds were released back into their original habitat at the sampling site. All procedures involving White-breasted Waterhens were reviewed and approved by the Faculty of Veterinary Science, Animal Care and Use Committee of Mahidol University (protocol no. MUVS-2020-11-55).

### 2.2. Staining and Microscopic Examination

Thin blood smears were stained with Giemsa’s azure–eosin–methylene blue solution (Merk KGaA, Darmstadt, Germany) in accordance with the manufacturer’s recommended protocol with phosphate buffer (pH 7.2) at 5% concentration for 1 h. Microscopic examinations of at least 100 fields were performed under low magnification (×400) for 5–10 min and high magnification (×1000) using a Nikon/ECIPSE Ti2 Inverted Microscope (Nikon Instruments Inc., New York, NY, USA). The intensity of infection was estimated from the number of infected erythrocytes per 10,000 cells [18]. The morphological and morphometric characteristics of the uninfected and infected erythrocytes and parasite gametocytes were imaged and measured using the NIS Elements AR 5.41.00 software (Nikon Instruments Inc., New York, NY, USA). *Haemoproteus* species were identified using the key morphologic characteristics based on “Keys to the avian *Haemoproteus* parasites” [17]. To estimate the difference in percentage of white blood cells (WBC) between infected and uninfected birds, the differential WBC count percentage was measured using blood smears of better quality following a previously reported protocol [19].

### 2.3. DNA Extraction and Mitochondrial Cytochrome b Gene Amplification

DNA was extracted from whole blood samples using the Genomic DNA Mini Kit (blood/cultured cells) (Geneaid Biotech, New Taipei City, Taiwan) in accordance with the manufacturer’s recommended protocol. The partial cytochrome *b* (*cytb*) gene was amplified using nested polymerase chain reaction (PCR), as previously described [20], with the outer primers HaemNFI and HaemNR3 and the inner primers HaemF and HaemR2. The initial PCR conditions were as follows: initial denaturation at 94 °C for 30 s; 20 cycles of amplification at 94 °C for 30 s, 50 °C for 30 s, and 72 °C for 45 s; and final extension at 72 °C for 10 min. The nested steps included the same PCRs with 35 cycles of amplification. The PCR reactions were performed in a 20 µL reaction volume containing 1 µL of DNA template or PCR product, 10 µL of 2 × PCR master mix solution (i-Taq™), 1 µL of each primer (10 µM), and up to 20 µL of ultrapure water. Ultrapure water and DNA samples extracted from positive-microscopy slides were used as negative and positive controls, respectively. Subsequently, 5 µL of the PCR product was used to determine the size of the amplicon by gel electrophoresis on 2% agarose gel using GelRed^®^ (Biotium, Fremont, CA, USA) and visualized using a BioSens SC-Series 710 gel documentation system (GenXpress, Wiener Neudorf, Austria). The positive PCR products were subjected to nucleotide sequencing based on the Sanger sequencing method by a commercial company (Bionics, Seoul, Republic of Korea) using both strands of inner primers. The electropherograms of all obtained sequences were carefully checked for double bases, which indicate co-infection, using BioEdit version 7.0.5.3 (Ibis Biosciences, Carlsbad, CA, USA) [21]. Sequences with ambiguous sites for multiple infections were rechecked using DnaSP version 6.12.03 [22]. Subsequently, the sequences were subjected to BLASTn searching using the NCBI GenBank and MalAvi databases [23]. The obtained nucleotides of *cytb* sequences with no match found to those previously published were considered to be a new lineage of haemosporidia and named according to the MalAvi nomenclature [23]. The obtained *cytb* sequences of all detected haemosporidian parasites were submitted and deposited to GenBank under accession numbers OQ565584–OQ565589.

### 2.4. Phylogenetic Analyses of the Partial Cytochrome b Gene Sequences

A phylogenetic tree was constructed to determine the relationships between the newly obtained molecular characteristics of *H. gallinulae* and other *Plasmodium* parasites and the previously reported morphospecies of haemosporidian parasites in Asia and Oceania, which is the habitat of the avian host White-breasted Waterhen. The alignment was constructed with 27 partial *cytb* sequences (479 bp) of well-known morphospecies of *Haemoproteus* and *Plasmodium* species, 13 individual lineages, and *Leucocytozoon majoris* (as the outgroup) using MAFFT version 7.520 software [24]. A Bayesian inference phylogeny was constructed using MrBayes version 3.2 software [25]. The best-fit substitution model was a general time-reversible model with gamma-distributed substitution rates and a proportion of invariant sites (GTR + I + G) selected using the Akaike information criterion in MrModeltest 2.4 software [26]. A Bayesian inference analysis was run for three million (×10^6^) generations, with sampling every hundredth generation. The first 25% of the tree was discarded as burn-in. The remaining 22,500 trees were calculated to determine the majority rule consensus tree, which was then visualized using FigTree.v1.4.4 [27]. The sequence divergence between sequences was calculated using MEGA X [28] with the Jukes–Cantor model [29].

### 2.5. Statistical Analysis

The heterophil-to-lymphocyte (H:L) ratio between uninfected and infected White-breasted Waterhens was compared using the Mann–Whitney U test. Comparisons of mean morphometric measurements between uninfected and infected erythrocytes as well as between macrogametocytes and microgametocytes were performed using Student’s independent *t*-test, after normal distribution and homogeneity of variance were confirmed using Shapiro–Wilk’s test and Levene’s test, respectively. A *p*-value of ≤0.05 was considered statistically significant.

## 3. Results

The overall prevalence of haemosporidian infection was 35% (6/17). No birds in this study had signs of pathogenicity. The differential WBC count of haemosporidian infection is shown in Table 1, although it was difficult to determine the stress indicator from the percentage of individual WBCs. However, the H:L ratio of differential WBCs was significantly higher (U = 27, *p* < 0.001) in infected White-breasted Waterhens than in uninfected ones (Table 1). Two birds were infected with a new lineage of the *Haemoproteus* parasite. However, the characteristic morphology of the new *Haemoproteus* lineage was identical to *H. gallinule* that was previously described in another host species, Slaty-legged Crake (*Rallina eurizonoides*) [15]. The intensity of parasitemia was extremely low, ranging 0.0001–0.003%. Four out of six partial *cytb* genes were amplified and the BLAST results were 100% identical to two known species: *P. elongatum* (lineage GRW06) and *P. collidatum* (lineage FANTAIL01) found in individual birds and an unknown *Plasmodium* species (GenBank no.: AB601445) found in two birds. Furthermore, the partial *cytb* sequences were deposited in GenBank under the accession numbers OQ565584–OQ565589 and one newly obtained sequence of *H. gallinulae* was assigned the lineage name AMPHO01 in the MalAvi database. Voucher specimen (accession no. G466274) of *H. gallinulae* from White-breasted Waterhens were deposited in the Queensland Museum, Queensland, Australia.

### 3.1. Morphological Characterization of Haemoproteus gallinulae de Mello, 1935 from Amaurornis phoenicurus in Thailand

**Figure 1:**
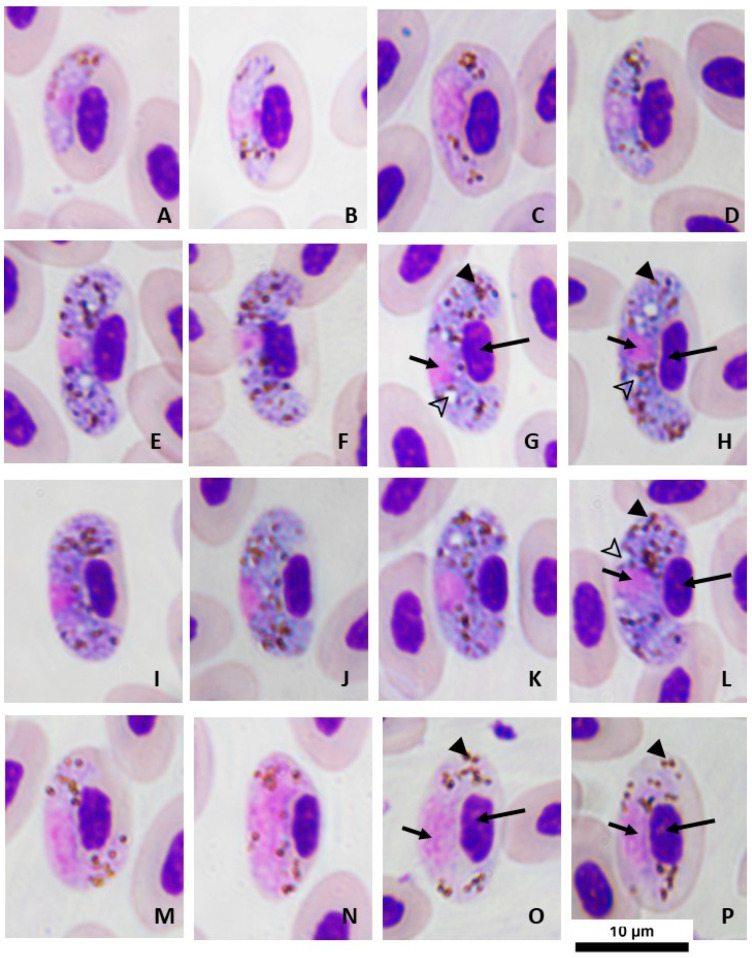
*Haemoproteus* (*Parahaemoproteus*) *gallinulae* from White-breasted Waterhens (*Amaurornis phoenicurus*) in Salaya suburb, Thailand. (**A**–**D**) Young gametocytes, (**E**–**L**) macrogametocytes, (**M**–**P**) microgametocytes. Long arrows—nuclei of infected erythrocytes; short arrows—nuclei of the parasite; arrow heads—pigment granules; outline arrow heads—vacuoles. Giemsa’s azure–eosin–methylene blue solution-stained thin blood films. Scale bar = 10 µm.

Young gametocytes (Table 2; Figure 1A–D): These were found in mature erythrocytes. The initial stage was found anywhere in the cytoplasm of infected erythrocytes. The developmental stage was usually found in a subpolar position relative to erythrocyte nuclei, touching neither nuclei nor the erythrocyte membrane. The outline of growing gametocytes (Figure 1B–D) showed an ameboid or irregular form covering both sides of the erythrocyte nuclei. Small vacuoles possibly appeared. Small-sized pigment granules (<0.5 µm) were disrupted or grouped in the cytoplasm, frequently found along the marginal outline.

Macrogametocytes (Table 2; Figure 1E–L): These were usually medium-sized and broadly halteridial, both sides of the marginal entire, usually covering the polar nucleus of erythrocyte infection. The cytoplasm was stained distinctly deep blue with Giemsa. Parasite nucleus was ovoid (almost round), stained pink, normally alongside the erythrocyte nucleus, closer to the erythrocyte membrane than to the erythrocyte nucleus (Figure 1E–L). Infected erythrocytes were significantly hypertrophied in all dimensions (*p* < 0.01). Erythrocyte nuclei were slightly atrophied (*p* < 0.05) in terms of the width and area but not in length (*p* > 0.05). Fully grown gametocytes distinctly laterally displaced the nuclei of the infected erythrocyte (Figure 1I–L; approximate nuclear displacement ratio [NDR] = 0.48). Pigment granules were small- to medium-sized (0.5–1 µm), ovoid, brownish, were approximately 30 in number, were generally scattered throughout the cytoplasm, and were possibly clumped in small group (3–4 granules).

Microgametocytes (Table 2; Figure 1M–P): These exhibited generally characteristic morphology of parasites, similar to macrogametocytes, with a distinct dimorphism in staining; however, fully grown microgametes almost completely covered both sides of the nuclear poles of infected erythrocytes (Figure 1N–P). Pigment granules were usually clumped into a cluster, scattered in the cytoplasm nearly marginal to the parasite nucleus, and were significantly less than those found in macrogametocytes. Erythrocytes infected by microgametocytes were distorted, similar to those infected by macrogametocytes (*p* > 0.05). The nuclei of infected erythrocytes were occasionally less displaced than those of macrogametocytes (*p* < 0.05).

### 3.2. Phylogenetic Relationships between Haemosporidian Parasites

**Figure 2:**
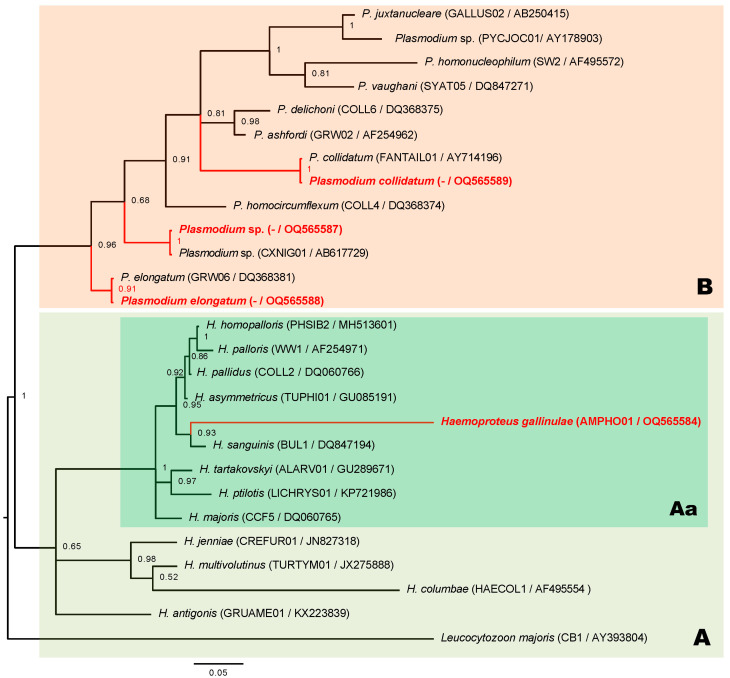
A Bayesian phylogeny of haemosporidian parasites infecting waterhens (*Amaurornis phoenicurus*) from Salaya suburb, Thailand. Clusters of genus *Haemoproteus* parasites (box A), subgenus *Parahaemoproteus* (box Aa) has *H. gallinulae* (red font) as member, and genus *Plasmodium* (box B) including three *Plasmodium* species (red font) obtained in this study; phylogenetic trees constructed based on partial sequences of *cytb* gene (27 sequences and 479 bp); values above the branches are posterior probabilities. *Leucocytozoon majoris* was an outgroup; parentheses show both MalAvi lineages and GenBank accession numbers.

Six out of seventeen White-breasted Waterhens tested positive for haemosporidian parasites. The same *cytb* sequences belonging to genus *Haemoproteus* (Figure 2, box A) which correspond morphospecies to *H. gallinulae* were detected in two of six individuals. This *Haemoproteus* species was a new lineage, namely, AMPHO01 (GenBank nos.: OQ565584 and OQ565585). Moreover, the other four sequences corresponded to three different lineages of *Plasmodium* parasites. Two sequences belonged to unidentified *Plasmodium* lineages (GenBank nos.: OQ565586 and OQ565587) and showed identity with the lineage CXNIG01. The other partial *cytb* sequence (GenBank no.: OQ565589) had 100% similarity to the *P. collidatum* lineage FANTAIL01, and the final sequence (GenBank no.: OQ565588) was closely related to *P. elongatum*. Only unique partial *cytb* sequences were included in the phylogeny (Figure 2). Coinfections by more than one parasite in the same individual were not detected.

The phylogenetic analysis revealed that the lineage of *H. gallinulae* AMPHO01 was significantly within the cluster of *Haemoproteus*-mediated *Culicoides* midges acting probably as a transmitting insect, indicating that this *Haemoproteus* species was likely a member of the subgenus *Parahaemoproteus* (Figure 2, box Aa). The genetic divergence among different lineages and between AMPHO01 and other lineages of the subgenus *Parahaemoproteus* ranged 9.6–12.7%. The close genetic distance of the *cytb* sequence-related AMPHO01 lineage was TUPHI01; the COLL2 and BUL1 lineages belonged to *H. asymmetricus*, *H. pallidus*, and *H. sanguinis*, the genetic distances for which were 9.6%, 9.6%, and 9.8%, respectively. However, these parasites were dissimilar in morphological features and host order between Gruiformes (Rallidae family) and Passeriformes. Furthermore, a closely related morphospecies of *H. gallinulae* was *H. antigonis*, belonging to the lineage GRUAME01, and described the only available molecular characteristics of Gruiformes birds. The genetic distance was 13.7% and likely higher than the lineage among the subgenus *Parahaemoproteus*. This indicated that the genetic distance of this *cytb* gene and the close relationship of *Haemoproteus* morphospecies found from Gruiformes birds were likely inconsistent. Regarding phylogeny, the *Plasmodium* lineage obtained in this study was clearly clustered individually (Figure 2, box B). The unidentified *Plasmodium* lineage (OQ565586) in this study was identical to *Plasmodium* lineage CXNIG01 and closely related to the *P. elongatum* GRW06 lineage, with a genetic distance of 4.3%, which was identical to the *Plasmodium* lineage (OQ565588) currently found. Another lineage of *Plasmodium* (OQ565589) was identical to *P. collidatum* FANTAIL01. The genetic divergence of lineage FANTAIL01 was related to *P. ashfordi* GRW02 with a genetic distance of 7.9%.

## 4. Discussion

This is the first report on haemosporidian species from White-breasted Waterhens in Thailand; furthermore, we herein report the molecular characteristics of *H. gallinulae* and describe its morphological characteristics in White-breasted Waterhens. Of the 15 Rallidae species found in Thailand, the White-breasted Waterhen is the only one surveyed for haemosporidian parasites [10]. Moreover, railbirds usually inhabit near wetlands, such as lowland agricultural fields. These locations have an abundance of insect vectors that positively influence haemosporidian infection [30]. Moreover, some Rallidae species were recorded to be residents in this region, including the Black-tailed Crake (*Porzana bicolor*), Grey-headed Swamphen (*Porphyrio poliocephalus*), Red-legged Crake (*Rallina fasciata*), and White-browed Crake (*Porzana cinerea*) or winter migratory species such as Baillon’s Crake (*Porzana pusilla*), Band-bellied Crake (*Porzana paykullii*), Common Moorhen (*Gallinula chloropus*), Eastern Water Rail (*Rallus indicus*), Eurasian Coot (*Fulica atra*), Ruddy-breasted Crake (*Porzana fusca*), Slaty-breasted Rail (*Gallirallus striatus*), Slaty-legged Crake (*Rallina eurizonoides*), Spotted Crake (*Porzana porzana*), and Watercock (*Gallicrex cinerea*) [10]. These migratory species can carry this parasite to other areas of their distribution. Therefore, this study provides potential information on haemosporidian parasites in railbirds, particularly White-breasted Waterhens. Furthermore, this study revealed the role of White-breasted Waterhens as a haemosporidian carrier such as a reservoir of avian malaria that involved a susceptible host of multi-avian host species. However, this study examined the haemosporidian parasite in regular adult birds. The pathogen infection possibly stimulated physiological stress [31]. The H:L ratio of differential WBC indicated stress [32,33]. Significantly high results were found in haemosporidian-infected birds (Table 1). This high ratio resulted in an increase in the number of heterophils. Owing to the limited sample size, this heterophil increase was possibly an observation of this parasite infection in this bird species. However, our study revealed haemosporidian infection in White-breasted Waterhens has certain limitations. For instance, the sample size might not fully represent the broader population of these birds. Additionally, potential differences related to sex and age of the birds were not explored in this study due to the limited data collected in these areas. Addressing these limitations in future research could provide more comprehensive insights into the epidemiology of haemosporidian infection in White-breasted Waterhens.

The results of this study revealed four different haemosporidian lineages. Only one was the *Haemoproteus* parasite for which the characteristic morphology was consistently identified to be *Haemoproteus gallinulae*. This *Haemoproteus* species was considered the first record in Thailand. Moreover, there were no data for GenBank accession and lineage code available for *H. gallinulae* [17]. Although this parasite had been previously characterized either in different host species from Slaty-legged Crake (*Rallina eurizonodes*) and American Coot (*Fulica americana*) [15] or type host, Common Moorhen (*Gallinula chloropus*) [34] exhibited a different characteristic morphology. This different morphology of *H. gallinulae* was hypothesized to depend on the other genera of hosts in the Rallidae family. In this study, the morphological features of the parasite were characterized in another host species, *Amaurornis phoenicurus* (Table 1). The effect of macro- and microgametocytes on erythrocyte distortion was consistent with previously reported findings [15]. Furthermore, *H. gallinulae* from White-breasted Waterhens presented the following key characteristics: (1) broadly halteridial form in fully grown gametocytes (Figure 1E–P); (2) markedly lateral displacement of the infected erythrocyte nuclei with an NDR of <0.5 (approximately 0.48 shown in macrogametocytes in the present study); and (3) number of pigment granules in macrogametocytes was >25 (about 29.89 in this study). These key features were consistent with the key characteristics of *Haemoproteus* parasite from Gruiformes birds [17]. However, a high pleomorphic form of *H. gallinulae* is likely represented in full-growing gametocytes, similar to those found in other *Haemoproteus* parasites such as *H. sacharovi* [35]. Therefore, the characteristic morphology of the *Haemoproteus* parasite presents the unique features of individual species, at least for all the key characteristic features that should be found in fully grown gametocytes.

The phylogeny of the haemosporidian parasite in White-breasted Waterhens revealed that *H. gallinulae* was distinctly classified into the subgenus *Parahaemoproteus*. Although the morphological characteristics between *H. gallinulae* and *H. antigonis* were indistinguishable, the details of pigment granules differed [17,36]. However, the genetic distance between these two morphospecies (13.7%) was higher than that between *H. asymmetricus* (9.6%) and *H. sanguinis* (9.8%), indicating that close genetic relationships exist between distinguishable morphospecies found in different hosts at the order level [37,38]. Similarly, there was a close genetic distance of the *cytb* sequence as well between *H. janniae* and *H. iwa,* but the morphospecies of these two *Haemoproteus* species are distinguishable [39,40]. Examination of only the genetic differences in the partial *cytb* sequence was not sufficient for distinguishing between some species of *Haemoproteus* parasites. Therefore, *Haemoproteus* species identification was performed based on morphological and molecular features.

Furthermore, the three obtained lineages of *Plasmodium* parasite in this study should be discussed. First, the *P. collidatum* (FANTAIL01) lineage was considered a generalist lineage that infects a wide range of hosts and had previously infected 16 bird species, including Rufous Fantail (*Rhipidura rufifrons*) [41,42]. However, this *Plasmodium* lineage had never been reported in White-breasted Waterhens. Subsequently, this Waterhen was added to one of several host species of the FANTAIL01 lineage in south and southeast Asia and the Oceania region [41,43]. Moreover, this lineage caused 25% mortality in experimental infection of Eurasian Siskin (*Spinus spinus*) [43]. This indicated that this lineage of *Plasmodium* parasite caused significantly virulent avian malaria.

Another *Plasmodium* lineage was the well-known *P. elongatum* GRW06 lineage, which is also presently found to infect White-breasted Waterhens. Although there are previous reports of infection in other species of the Rallidae family, such as Corncrake (*Crex crex*) and Purple Gallinule (*Porphyrio martinica*), this *Plasmodium* lineage was distributed worldwide in resident and migratory birds such as Great Reed-warbler (*Acrocephalus arundinaceus*), Madagascar Swamp Warbler (*Acrocephalus newtoni*), Eurasian Marsh-Harrier (*Circus aeruginosus*), and Carrion Crow (*Corvus corone*) [44,45,46,47], together with marine birds such as penguin (*Spheniscus magellanicus*) [48]. Furthermore, infection with *P. elongatum* GRW06 was a cause of severe avian malaria [49]. Another *Plasmodium* lineage had not been identified at the species level belonging to the CXNIG01 lineage that was isolated from *Culex nigropunctatus* mosquitoes in Japan [50]. Moreover, this unidentified *Plasmodium* (GenBank no.: AB601445) was previously reported to infect White-breasted Waterhens. This lineage of *Plasmodium* parasite was rarely recorded in islands of Japan. Therefore, to the best of our knowledge, this is the first report of this *Plasmodium* lineage in additional geographic regions in Salaya suburb, Thailand.

## 5. Conclusions

This is the first study of haemosporidian infection in White-breasted Waterhen resident birds. The findings present a new record of haemosporidian parasites in Thailand, and the *H. gallinulae* AMPHO01 lineage was characterized morphologically from different host species in Rallidae. Phylogenetic analysis revealed close lineages of *Haemoproteus* species classified into the subgenus *Parahaemoproteus*. Although morphospecies between *H. gallinulae* and *H. antigonis* (*Haemoproteus* of Gruiformes host) were difficult to distinguish, the genetic distance of partial *cytb* sequences was probably distinctly different. Moreover, this study found three different lineages of *Plasmodium* parasite. Although one of them was not identified as a species found in *Culex* mosquitoes, it was found in the avian host White-breasted Waterhen. Furthermore, two lineages, namely, *P. collidatum* and *P. elongatum*, were identified for the first time in this avian host. These findings reveal the role of White-breasted Waterhens in possibly harboring multiple species or serving as a reservoir host of the haemosporidian parasite. Knowledge of the relationship between the host and haemosporidian parasites can provide a principle for disease management.

## Figures and Tables

**Table 1:** Comparison of differential white blood cell (WBC) counts and heterophil-to-lymphocyte (H:L) ratio in White-breasted Waterhens (*Amaurornis phoenicurus*) from representative four of eleven uninfected (negative) and six infected (positive) with haemosporidian parasites. Data are expressed as mean and range (in parentheses).

	Haemosporidian Infection Status	
	Negative (n = 4)	Positive (n = 6)
WBC (×10³ cells/mm³)	5.2 (3.9–6.6)	7.2 (6.2–8.4)
Heterophils (%)	59.3 (54.0–67.0)	64.8 (53.0–74.0)
Lymphocytes (%)	37.3 (23.0–44.0)	31.3 (21.0–44.0)
Monocytes (%)	3.5 (1.0–10.0)	3.8 (2.0–8.0)
H:L ratio	1.7 (1.2–2.9)	2.3 (1.2–3.5)

**Table 2:** Morphometry of *Haemoproteus gallinulae* de Mello, 1935, in White-breasted Waterhens (*Amaurornis phoenicurus*) from Salaya suburb, Thailand.

Measurements	Uninfected Erythrocyte		Macrogametocyte		Microgametocyte	
	(n = 28)		(n = 37)		(n = 28)	
	Range	Mean ± SD	Range	Mean ± SD	Range	Mean ± SD
Erythrocyte						
Length	13.49–12.13	13.02 ± 0.38	12.59–15.92	14.29 ± 0.73	12.84–15.86	14.27 ± 0.78
Width	8.14–6.52	7.28 ± 0.44	6.36–8.48	7.68 ± 0.54	6.80–8.54	7.65 ± 0.47
Area	83.42–66.34	74.29 ± 4.97	74.42–101.11	86.99 ± 6.39	76.73–97.88	85.71 ± 5.88
Erythrocyte nucleus						
Length	5.1–6.59	5.77 ± 0.35	5.00–6.46	5.66 ± 0.36	4.49–6.37	5.64 ± 0.44
Width	2.67–3.72	3.02 ± 0.21	2.35–3.18	2.78 ± 0.19	2.22–3.21	2.78 ± 0.23
Area	10.8–16.28	14.21 ± 1.17	11.58–16.16	13.58 ± 1.14	10.56–17.61	13.41 ± 1.64
NDR *	-	-	0.11–0.92	0.48 ± 0.22	0.31–0.98	0.62 ± 0.16
Gametocyte						
Length	-	-	12.22–15.27	13.61 ± 0.78	12.08–15.02	13.16 ± 0.80
Width	-	-	2.16–5.30	3.66 ± 0.59	2.57–4.12	3.28 ± 0.38
Area	-	-	41.12–67.75	56.19 ± 6.88	39.47–61.55	49.69 ± 5.34
Pigment granules	-	-	13–40	29.89 ± 5.79	17–27	21.70 ± 2.70
Gametocyte nucleus						
Length	-	-	2.08–5.26	3.33 ± 0.79	4.52–8.56	6.46 ± 0.94
Width	-	-	1.92–4.03	2.86 ± 0.40	1.81–3.71	2.79 ± 0.46
Area	-	-	5.16–12.45	8.14 ± 1.76	10.45–20.08	15.47 ± 2.78

* Nuclear displacement ratio according to Bennett and Campbell (1972).

## Data Availability

The partial cytochrome (*cytb*) gene sequences have been deposited in GenBank under the accession numbers OQ565584 to OQ565589. Voucher specimens (accession no. G466274) of *H. gallinulae* from White-breasted Waterhens were deposited in the Queensland Museum, Queensland, Australia.

## References

[1] Valkiunas G. (2004). Avian Malaria Parasites and Other Haemosporidia.

[2] Sol D., Jovani R., Torres J. (2003). Parasite mediated mortality and host immune response explain age-related differences in blood parasitism in birds. Oecologia.

[3] Garcia-Longoria L., Palinauskas V., Ilgūnas M., Valkiūnas G., Hellgren O. (2020). Differential gene expression of *Plasmodium homocircumflexum* (lineage pCOLL4) across two experimentally infected passerine bird species. Genomics.

[4] Marzal A., Reviriego M., Hermosell I.G., Balbontín J., Bensch S., Relinque C., Rodríguez L., Garcia-Longoria L., de Lope F. (2013). Malaria infection and feather growth rate predict reproductive success in house martins. Oecologia.

[5] Coon C.A.C., Garcia-Longoria L., Martin L.B., Magallanes S., de Lope F., Marzal A. (2016). Malaria infection negatively affects feather growth rate in the house sparrow Passer domesticus. J. Avian Biol..

[6] Palinauskas V., Žiegytė R., Ilgūnas M., Iezhova T.A., Bernotienė R., Bolshakov C., Valkiūnas G. (2015). Description of the first cryptic avian malaria parasite, *Plasmodium homocircumflexum* n. sp., with experimental data on its virulence and development in avian hosts and mosquitoes. Int. J. Parasitol..

[7] Gulliver E., Hunter S., Howe L., Castillo-Alcala F. (2022). The pathology of fatal avian malaria due to *Plasmodium elongatum* (GRW6) and *Plasmodium matutinum* (LINN1) infection in New Zealand kiwi (*Apteryx* spp.). Animals.

[8] Schumm Y.R., Metzger B., Neuling E., Austad M., Galea N., Barbara N., Quillfeldt P. (2021). Year-round spatial distribution and migration phenology of a rapidly declining trans-Saharan migrant—Evidence of winter movements and breeding site fidelity in European turtle doves. Behav. Ecol. Sociobiol..

[9] Zehtindjiev P., Križanauskienė A., Bensch S., Palinauskas V., Asghar M., Dimitrov D., Scebba S., Valkiūnas G. (2012). A new morphologically distinct avian malaria parasite that fails detection by established polymerase chain reaction-based protocols for amplification of the cytochrome *B* gene. J. Parasitol..

[10] Round P. Checklist of the Birds of Thailand. https://www.thaibirding.com/book_reviews/roundlist.htm.

[11] Kumar P., Gupta S.K. (2009). Diversity and abundance of wetland birds around Kurukshetra, India. Our Nat..

[12] Bhaibulaya M., Indra-Ngarm S., Ananthapruti M. (1979). Freshwater fishes of Thailand as experimental intermediate hosts for *Capillaria philippinensis*. Int. J. Parasitol..

[13] Yoshino T., Uemura J., Endoh D., Kaneko M., Osa Y., Asakawa M. (2009). Parasitic nematodes of anseriform birds in Hokkaido, Japan. Helminthologia.

[14] Jaafar N., Babjee M.A. (2011). Parasites of the White-Breasted Waterhen (Amaurornis phoenicurus).

[15] Bennett G.F. (1980). Avian Haemoproteidae. 14. The haemoproteids of the avian family Rallidae. Can. J. Zool..

[16] Prompiram P., Kaewviyudth S., Sukthana Y., Rattanakorn P. (2015). Study of morphological characteristic and prevalence of haemoproteus blood parasite in passerines in bung Boraphet. Thai J. Vet. Med..

[17] Valkiūnas G., Iezhova T.A. (2022). Keys to the avian *Haemoproteus* parasites (Haemosporida, Haemoproteidae). Malar. J..

[18] Godfrey R.D., Fedynich A.M., Pence D.B. (1987). Quantification of hematozoa in blood smears. J Wildl. Dis..

[19] Clark P., Boardman W., Raidal S. (2009). Atlas of Clinical Avian Hematology.

[20] Waldenström J., Bensch S., Hasselquist D., Ostman O. (2004). A new nested polymerase chain reaction method very efficient in detecting *Plasmodium* and *Haemoproteus* infections from avian blood. J. Parasitol..

[21] Hall T.A. (1999). BioEdit: A User-Friendly Biological Sequence Alignment Editor and Analysis Program for Windows 95/98/NT.

[22] Rozas J., Ferrer-Mata A., Sánchez-DelBarrio J.C., Guirao-Rico S., Librado P., Ramos-Onsins S.E., Sánchez-Gracia A. (2017). DnaSP 6: DNA sequence polymorphism analysis of large data sets. Mol. Biol. Evol..

[23] Bensch S., Hellgren O., Pérez-Tris J. (2009). Malavi MalAvi: A public database of malaria parasites and related haemosporidians in avian hosts based on mitochondrial cytochrome b lineages. Mol. Ecol. Resour..

[24] Katoh K., Rozewicki J., Yamada K.D. (2019). MAFFT online service: Multiple sequence alignment, interactive sequence choice and visualization. Brief Bioinform..

[25] Ronquist F., Huelsenbeck J.P. (2003). MrBayes 3: Bayesian phylogenetic inference under mixed models. Bioinformatics.

[26] Nylander J.A.A. (2004). MrModelTest v2. Program Distributed by the Author: Evolutionary Biology Centre.

[27] Rambout A. FigTree: Tree Figure Drawing Tool, Version 1.4.0. http://tree.bio.ed.ac.uk/software/figtree/.

[28] Kumar S., Stecher G., Li M., Knyaz C., Tamura K. (2018). MEGA X: Molecular evolutionary genetics analysis across computing platforms. Mol. Biol. Evol..

[29] Jukes T.H., Cantor C.R. (1969). Evolution of Protein Molecules.

[30] Van Hoesel W., Marzal A., Magallanes S., Santiago-Alarcon D., Ibáñez-Bernal S., Renner S.C. (2019). Management of ecosystems alters vector dynamics and haemosporidian infections. Sci. Rep..

[31] Davis A.K., Maney D.L., Maerz J.C. (2008). The use of leukocyte profiles to measure stress in vertebrates: A review for ecologists. Funct. Ecol..

[32] Gross W.B., Siegel H.S. (1983). Evaluation of the heterophil/lymphocyte ratio as a measure of stress in chickens. Avian Dis..

[33] Maxwell M.H. (1993). Avian blood leucocyte responses to stress. Worlds Poult. Sci. J..

[34] Sacchi L., Prigioni C. (1986). Haematozoa of Italian birds. II. First European record of *Haemoproteus gallinulae* de Mello, 1935. Gallinula chloropus and Redescription (Apicomplexa Haemosporina).

[35] Valkiūnas G., Santiago-Alarcon D., Levin I.I., Iezhova T.A., Parker P.G. (2010). A new *Haemoproteus* species (Haemosporida: Haemoproteidae) from the endemic Galapagos dove *Zenaida galapagoensis*, with remarks on the parasite distribution, vectors, and molecular diagnostics. J. Parasitol..

[36] Bertram M.R., Hamer S.A., Hartup B.K., Snowden K.F., Medeiros M.C., Outlaw D.C., Hamer G.L. (2017). A novel Haemosporida clade at the rank of genus in North American cranes (Aves: Gruiformes). Mol. Phylogenet. Evol..

[37] Musa S., Mackenstedt U., Woog F., Dinkel A. (2022). Untangling the actual infection status: Detection of avian haemosporidian parasites of three Malagasy bird species using microscopy, multiplex PCR, and nested PCR methods. Parasitol. Res..

[38] Valkiūnas G., Ilgūnas M., Bukauskaitė D., Duc M., Iezhova T.A. (2021). Description of *Haemoproteus asymmetricus* n. sp. (Haemoproteidae), with remarks on predictability of the DNA haplotype networks in haemosporidian parasite taxonomy research. Acta Trop..

[39] Levin I.I., Valkiūnas G., Iezhova T.A., O’Brien S.L., Parker P.G. (2012). Novel *Haemoproteus* species (Haemosporida: Haemoproteidae) from the swallow-tailed gull (Lariidae), with remarks on the host range of hippoboscid-transmitted avian hemoproteids. J. Parasitol..

[40] Levin I.I., Valkiūnas G., Santiago-Alarcon D., Cruz L.L., Iezhova T.A., O’Brien S.L., Hailer F., Dearborn D., Schreiber E.A., Fleischer R.C. (2011). Hippoboscid-transmitted *Haemoproteus* parasites (Haemosporida) infect Galapagos Pelecaniform birds: Evidence from molecular and morphological studies, with a description of *Haemoproteus iwa*. Int. J. Parasitol..

[41] Beadell J.S., Gering E., Austin J., Dumbacher J.P., Peirce M.A., Pratt T.K., Atkinson C.T., Fleischer R.C. (2004). Prevalence and differential host-specificity of two avian blood parasite genera in the Australo-Papuan region. Mol. Ecol..

[42] Silva-Iturriza A., Ketmaier V., Tiedemann R. (2012). Profound population structure in the Philippine Bulbul *Hypsipetes philippinus* (Pycnonotidae, Aves) is not reflected in its *Haemoproteus* haemosporidian parasite. Infect. Genet. Evol..

[43] Platonova E., Aželytė J., Iezhova T., Ilgūnas M., Mukhin A., Palinauskas V. (2021). Experimental study of newly described avian malaria parasite *Plasmodium* (*Novyella*) *collidatum* n. sp., genetic lineage pFANTAIL01 obtained from South Asian migrant bird. Malar. J..

[44] Pérez-Tris J., Hellgren O., Krizanauskiene A., Waldenström J., Secondi J., Bonneaud C., Fjeldså J., Hasselquist D., Bensch S. (2007). Within-host speciation of malaria parasites. PLoS ONE.

[45] Harl J., Himmel T., Valkiūnas G., Ilgūnas M., Nedorost N., Matt J., Kübber-Heiss A., Alic A., Konicek C., Weissenböck H. (2022). Avian haemosporidian parasites of accipitriform raptors. Malar. J..

[46] Harl J., Himmel T., Valkiūnas G., Weissenböck H. (2019). The nuclear 18S ribosomal DNAs of avian haemosporidian parasites. Malar. J..

[47] Musa S., Mackenstedt U., Woog F., Dinkel A. (2019). Avian malaria on Madagascar: Prevalence, biodiversity and specialization of haemosporidian parasites. Int. J. Parasitol..

[48] Vanstreels R.E.T., Gardiner C.H., Yabsley M.J., Swanepoel L., Kolesnikovas C.K.M., Silva-Filho R.P., Ewbank A.C., Catão-Dias J.L. (2018). Schistosomes and microfilarial parasites in Magellanic penguins. J. Parasitol..

[49] Palinauskas V., Žiegytė R., Iezhova T.A., Ilgūnas M., Bernotienė R., Valkiūnas G. (2016). Description, molecular characterisation, diagnostics and life cycle of *Plasmodium elongatum* (lineage pERIRUB01), the virulent avian malaria parasite. Int. J. Parasitol..

[50] Ejiri H., Sato Y., Kim K.S., Tamashiro M., Tsuda Y., Toma T., Miyagi I., Murata K., Yukawa M. (2011). First record of avian *Plasmodium* DNA from mosquitoes collected in the Yaeyama Archipelago, southwestern border of Japan. J. Vet. Med. Sci..

